# Lance–Adams syndrome or chronic post-hypoxic myoclonus in adults: a systematic literature review

**DOI:** 10.1093/braincomms/fcaf329

**Published:** 2025-09-09

**Authors:** Geoffroy Vellieux, Emmanuelle Apartis, Vincent Navarro

**Affiliations:** Paris Brain Institute, ICM, Inserm, CNRS, Sorbonne Université, Paris F-75013, France; Assistance Publique-Hôpitaux de Paris, EEG Unit, Department of Neurology, Pitié-Salpêtrière Hospital, Paris F-75013, France; Paris Brain Institute, ICM, Inserm, CNRS, Sorbonne Université, Paris F-75013, France; Assistance Publique-Hôpitaux de Paris, Neurophysiology of Movement Disorders Unit, Department of Neurophysiology, Saint-Antoine and Pitié-Salpêtrière Hospitals, Paris F-75012 and F-75013, France; Paris Brain Institute, ICM, Inserm, CNRS, Sorbonne Université, Paris F-75013, France; Assistance Publique-Hôpitaux de Paris, EEG Unit, Department of Neurology, Pitié-Salpêtrière Hospital, Paris F-75013, France; Assistance Publique-Hôpitaux de Paris, Epilepsy Unit, Department of Neurology, Reference Center of Rare Epilepsies, ERN-EpiCare, Pitié-Salpêtrière Hospital, Paris F-75013, France

**Keywords:** myoclonus, Lance–Adams syndrome, post-hypoxic myoclonus, cardiorespiratory arrest, motor cortex

## Abstract

Lance–Adams syndrome, or chronic post-hypoxic myoclonus, is a disabling chronic myoclonic disorder occurring in survivors of brain hypoxic events. Using a systematic methodology for literature search and data acquisition, we extensively reviewed all published cases of Lance–Adams syndrome since the first original patients were described by JW Lance and RD Adams in 1963. We analysed the available data of 272 patients extracted from the 153 included studies to summarize the natural history of Lance–Adams syndrome, outline the full spectrum of this disorder and deepen our understanding of its underlying mechanisms. Two-thirds of patients suffered from a respiratory hypoxic leading event. The main causes of anoxia were peri-surgery and anaesthetic accidents, asthma attacks/bronchospasm, cardiac disorders and intoxications/drug overdoses. Many patients exhibited a ‘pure’ Lance–Adams syndrome, characterized by multi-focal action-induced myoclonic jerks predominant to distal limbs. Morphologic brain imaging did not show any specific abnormalities. Neurophysiological evaluations, including EEG recordings, polymyography of myoclonus and jerk-locked back averaging of myoclonus, revealed features of cortical myoclonus in the majority of patients. Both EEG, showing epileptiform discharges on the frontal and central median regions, and brain metabolism imaging, showing hypometabolism on the pericentral regions, indicated that myoclonus in Lance–Adams syndrome originates within the motor cortex. Some anti-seizure medications have shown some effectiveness and certain neuromodulation techniques have promising effects.

## Teaser

Vellieux *et al*. compile all the available data about Lance–Adams syndrome to elucidate the natural history, outline the full spectrum and deepen our understanding of this disorder. They provide new perspectives on the mechanistic underpinnings of Lance–Adams syndrome, particularly regarding the cortical or sub-cortical origin of myoclonus.

## Graphical Abstract

**Graphical Abstract:**
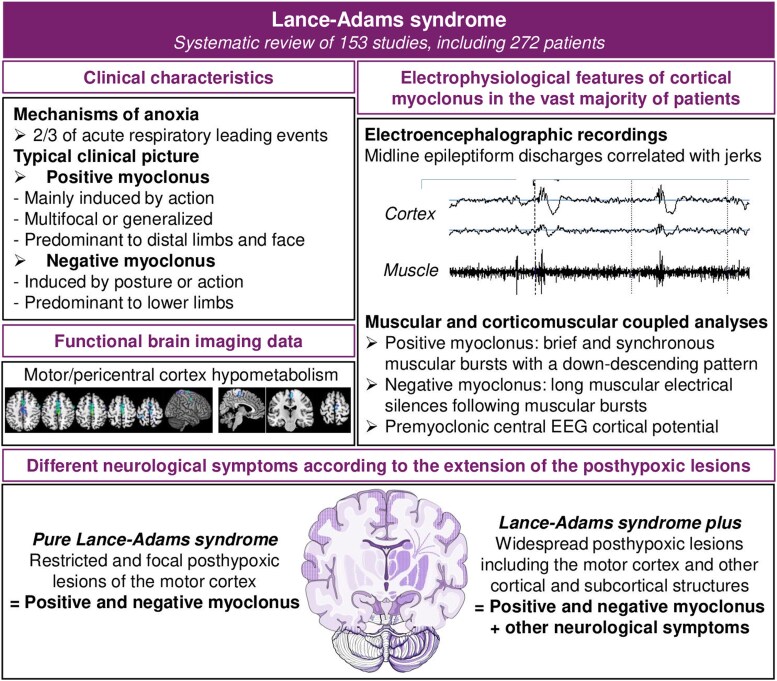

## Introduction

Chronic post-hypoxic myoclonus (PHM) is a severe disorder in survivors of brain hypoxic events. The first patients were described in detail by JW Lance and RD Adams in 1963.^1,2^ They reported four patients who developed myoclonic jerks particularly initiated by intention and voluntary movements after a post-anoxic coma. They gave the term ‘intention or action myoclonus’ to this syndrome, as a result of hypoxic encephalopathy. Since then, many other reports have been published under the eponymous Lance–Adams syndrome (LAS). Nowadays, LAS is defined only by clinical features, as a multi-focal or generalized, intention/action or stimulus-induced myoclonus occurring after an anoxic insult when patients regain consciousness.^3,4^

The current literature on LAS mainly consists of case reports or little case series. Many data have been reported covering various fields of this disorder. Besides their interest in helping physicians to manage patients, data on LAS may help better understand its pathophysiological mechanisms, especially the myoclonus generator’s cortical and/or sub-cortical localization.

We aimed to systematically collect data available on patients with LAS to draw the natural history of this disorder, summarize the whole spectrum of this disabling chronic disorder and gain deeper insights into its underlying mechanisms.

## Methodology

We searched for studies published from 1963 to 1 September 2024. This review adhered to the Preferred Reporting Items for Systematic Reviews and Meta-Analyses (PRISMA) guidelines.^5^ We conducted a literature search on the PubMed database using the terms ‘Lance–Adams syndrome’ (search no. 1), ‘posthypoxic myoclonus’ (search no. 2), ‘postanoxic myoclonus’ (search no. 3), ‘post-hypoxic myoclonus’ (search no. 4), ‘post-anoxic myoclonus’ (search no. 5), ‘post hypoxic myoclonus’ (search no. 6) and ‘post anoxic myoclonus’ (search no. 7). We removed duplicate articles and studies published in another language than English or French. We screened the titles and abstracts for articles relevant to our review. We excluded studies reporting perinatal anoxia or paediatric patients and pre-clinical data or animal models. We did not include the studies for which we could not retrieve the full text. After the full-text screening of retrieved reports, we excluded articles reporting patients with insufficient data to support a diagnosis of LAS (i.e. no clear history of hypoxia/anoxia or ischaemia prior to myoclonus or no persistent movement disorders clearly identified as myoclonic jerks in patients whose level of consciousness allowed for a minimal examination of myoclonus), articles reporting patients with acute PHM/status myoclonus/myoclonus status epilepticus (SE) who did not develop a chronic state of myoclonus suitable with LAS or patients with early death, articles dealing with PHM without reports of originally described patients with LAS, articles reporting large cohorts of patients including patients with LAS but without individual precise data about these patients and articles dealing with another topic than LAS. Finally, we screened the reference lists of eligible reports and reviews addressing PHM^3,4,6,7^ and included relevant articles that were missed by our PubMed searches and that fitted with our inclusion criteria.

For each included study, comprehensive data were extracted regarding (i) characteristics of the anoxic leading event; (ii) clinical features, especially characteristics of myoclonus and other neurological symptoms; (iii) biological data, including blood and CSF; (iv) anatomopathological findings with post-mortem human explorations; (v) neurophysiological characteristics, especially EEG recordings, polygraphic EMG recordings of myoclonus, EEG–EMG coupled analyses such as EEG jerk-locked back averaging (JLBA), somatosensory evoked potentials (SSEP) and long latency reflexes (LLRs); (vi) brain imaging findings, including morphologic MRI and functional explorations such as PET, single photon emission computed tomography (SPECT), functional MRI (fMRI) and magnetic resonance spectroscopy (MRS) and (vii) pharmacological and non-pharmacological treatments. Data were collected systematically using the same customized spreadsheet for each patient. We were careful not to include duplicate data of patients reported in different studies.

A Shapiro–Wilk normality test was performed for each numerical parameter. Normal data are presented as mean ± standard deviation and non-normal data as median (min−max).

## Selected studies

**Figure 1:**
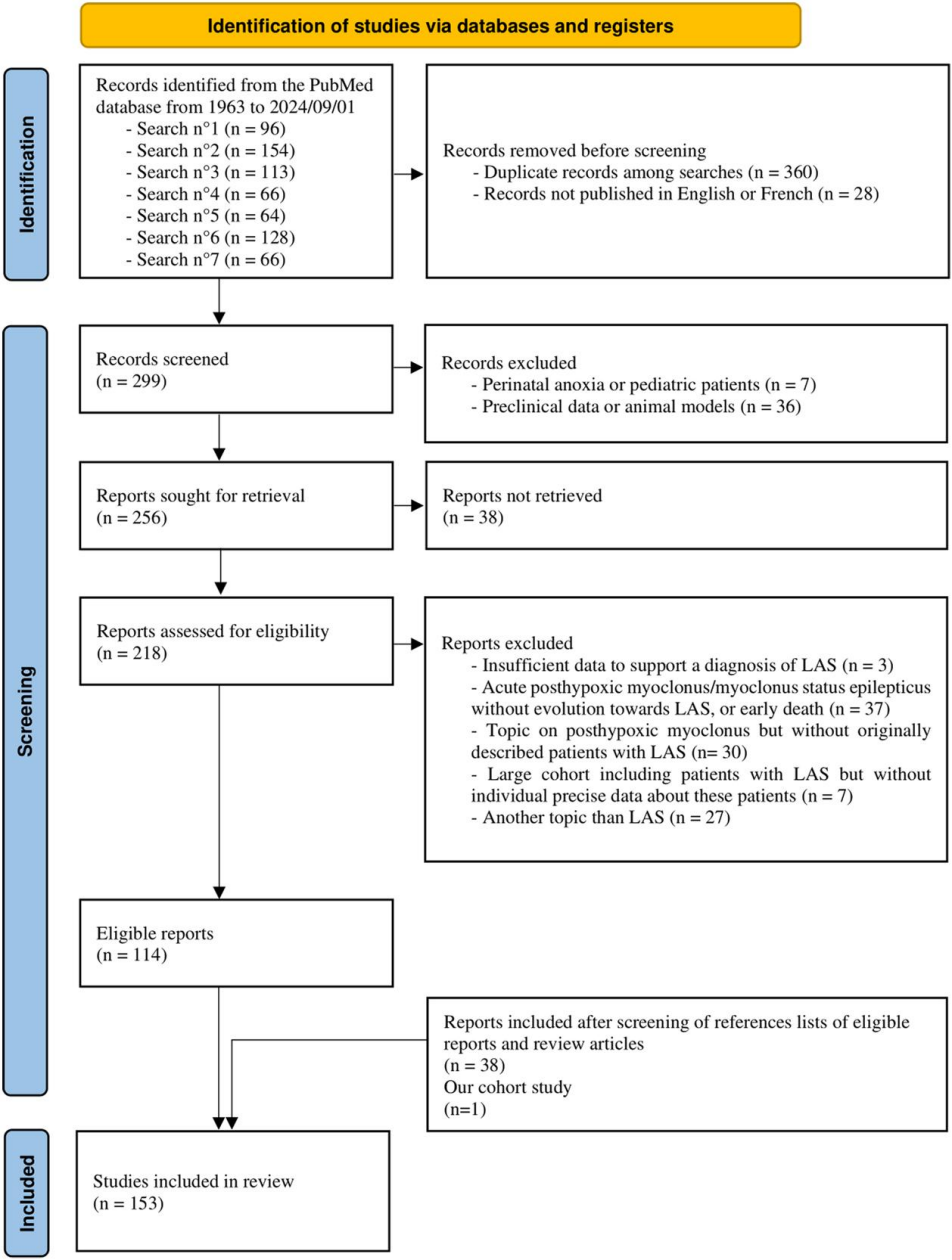
PRISMA flowchart.

The PRISMA flow diagram is in Fig. 1. All articles identified, screened, excluded and included in this review are available in Table S1. We initially identified 687 articles. We removed 388 articles because they were duplicate records or not published in English or French. After screening the remaining 299 records, we obtained 114 eligible reports and added 38 other studies from reference lists and our cohort study.^8^ Thus, we included 153 studies, mostly case reports, for a total of 272 patients. The number of patients who benefited from careful and extended explorations was small. We identified many missing or imprecise data, so we will most often report the number of patients with precise data available for the variable under study. For example, for the sex of the 272 patients included in this review, the data were available (DA) in 247 and further reported as ‘DA 247/272’. Sex was distributed in 57% of men (141 out of 247) and 43% of women (106 out of 247).

## General characteristics of the anoxic leading event

### Mechanisms and aetiologies — Lance–Adams syndrome is mainly associated with acute hypoxic respiratory events

**Figure 2:**
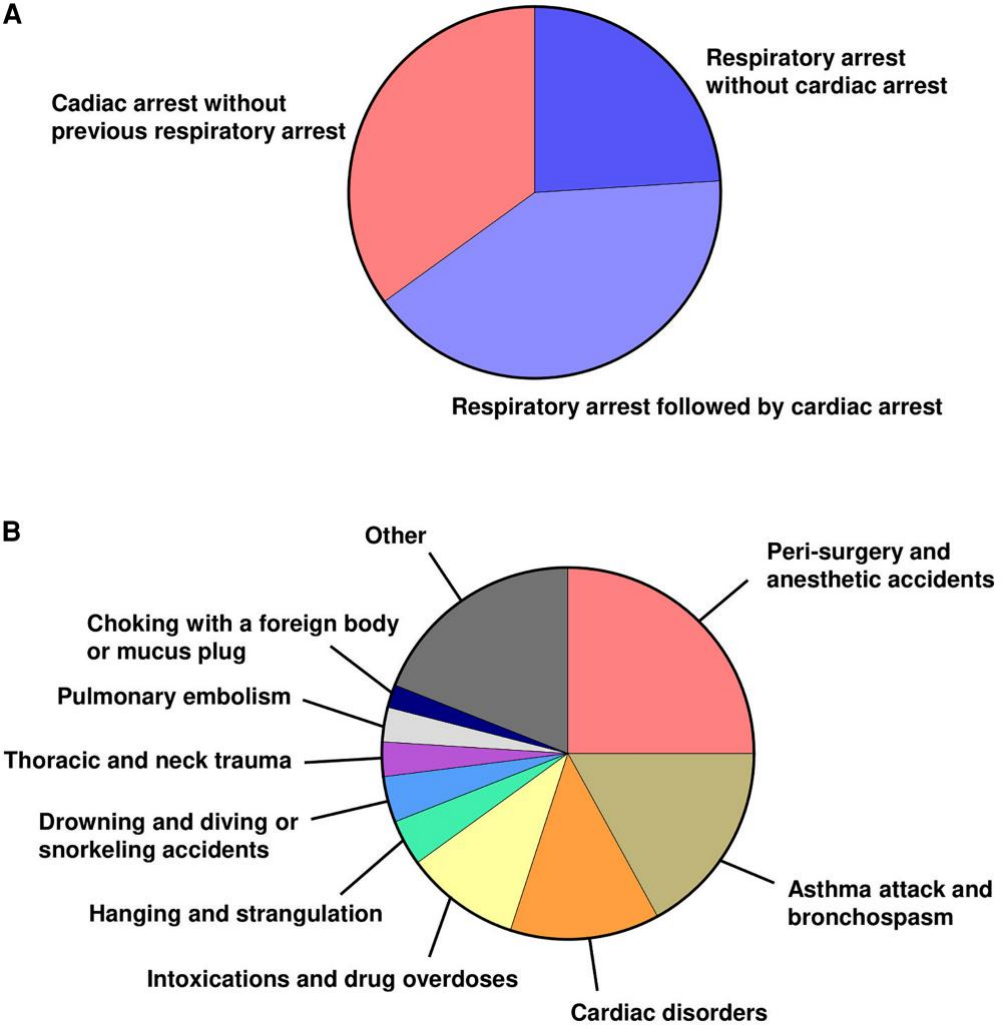
**Mechanisms and aetiologies of anoxia in patients with LAS.** (**A**) Mechanisms of anoxia in 192/272 patients. (**B**) Aetiologies of the leading anoxic event in 216/272 patients.

The anoxic event leading to LAS occurred at 43 (15–83) years old (DA 249/272). A respiratory arrest/failure without cardiac arrest (CA) was reported in 24%, and a CA following a previous respiratory arrest/failure was reported in 41% (DA 192/272, Fig. 2A). Thus, two-thirds of patients with LAS suffered from a primary hypoxic respiratory event, leading or not to CA. The causes of the anoxic event were various (DA 216/272, Fig. 2B). The leading aetiology was peri-surgery/anaesthetic accidents, found in 25% of patients. In most cases, authors did not specify a precise mechanism but rarely reported intubation/extubation issues, upper airway compression, cardiac tamponade, coronary artery trauma or laryngitis/laryngeal oedema. The second main cause was asthma attack/bronchospasm, found in 17% of patients, rarely associated with pneumothorax, allergic/anaphylactic reaction, pneumonia/bronchitis or laryngitis/laryngeal oedema. Cardiac disorders were the third main cause, in 13% of patients, mainly myocardial infarction, and occasionally arrhythmia, myocarditis, cardiomyopathy and myocardial stress test. Intoxications and drug overdoses were reported in 10% of patients. Less frequently, we found hanging/strangulation (4%), drowning or diving/snorkeling accidents (4%), thoracic and neck trauma (3%), pulmonary embolism (3%) and choking with a foreign body or mucus plug (2%). These data are in contrast with those reported in other critically ill patients with hypoxic-ischaemic brain injury, which showed a large predominance of cardiac causes.^9-11^ This discrepancy may reflect a unique susceptibility of LAS to non-cardiac hypoxic insults or, alternatively, a selection bias since only patients who survive non-cardiac hypoxia can develop this chronic myoclonus syndrome.

No-flow and low-flow periods were rarely reported among patients with CA. However, among all patients included, the total duration of the anoxic event was 15 (1–85) minutes (DA 90/272). The post-anoxic coma had a duration of 9 (0–48) days (DA 130/272).

## Clinical features of myoclonus

The overall onset delay of myoclonus in included patients with LAS was 3 (0.2–90) days after the anoxic event (DA 121/272, irrespective of two patients reporting a starting point of myoclonus many years after anoxia^8,12^). Myoclonus appeared during coma in 38% and after regaining consciousness/sedatives weaning in 62% (DA 105/272). We will first present the features of myoclonus in patients during coma and then the features of myoclonus after the coma period/regaining consciousness for all patients with LAS. The data about myoclonus’ localization and activation mode presented thereafter are non-mutually exclusive.

### Characteristics of myoclonus during the coma period of patients who will further develop Lance–Adams syndrome — Spontaneous and reflex generalized myoclonus may appear during coma and convert to action-induced multi-focal or generalized myoclonus after regaining consciousness

Among patients with LAS who exhibited their first myoclonic jerks during the coma period, myoclonus was stimuli-induced in 36%, spontaneous in 32%, and both spontaneous and stimuli-induced in 32% (DA 25/40) during coma. However, after these patients regained consciousness, jerks were mainly present during action in all (DA 40/40). Moreover, jerks during coma were generalized in 79% and multi-focal in 21% (DA 29/40). However, after these patients regained consciousness, jerks were multi-focal in 37%, generalized in 37%, and both generalized and multi-focal in 26% (DA 35/40).

LAS is classically associated with myoclonus after regaining consciousness. However, these data suggest that a few patients who will further develop LAS may previously show generalized spontaneous and reflex myoclonus during coma, changing to multi-focal action myoclonus predominant to the limbs thereafter.

The diagnosis of LAS is nevertheless challenging in comatose patients. Some authors define as ‘acute’ PHM any patient with myoclonus’ onset during the first hours or days after the anoxic event. The prognosis is heterogeneous among these patients. Some ‘acute’ PHM can be related to myoclonus SE, mainly characterized by generalized myoclonus and malignant generalized EEG patterns. These are related to severe anoxic-ischemic brain injuries and are usually associated with a poor outcome.^13^ However, some other patients with ‘acute’ PHM may then develop LAS, for some defined as ‘chronic’ PHM after regaining consciousness, portending a far better prognosis, even if they presented with generalized myoclonus in the comatose post-anoxic period. Moreover, sedatives may delay the myoclonus onset considering that some patients presented their first myoclonic jerks just after the sedatives weaning. The use of some neurophysiological biomarkers, particularly the EEG pattern (described in the below-dedicated section), may help intensivists in the neurological prognostication of patients with myoclonus during coma.^14,15^ The early onset of PHM does not invariably predict a poor outcome related to dramatic anoxic-ischemic brain injuries.^16,17^ The identification of patients with myoclonus during coma who might survive and progress to LAS may thus prevent discontinuation of ventilatory support and withdrawal of life-sustaining therapies due to inaccurate prognostication.

### Characteristics of myoclonus after the coma period/regaining consciousness — The main features of Lance–Adams syndrome are action-induced multi-focal myoclonic jerks predominant to distal limbs associated with lower limbs negative myoclonus

Far from the coma period, myoclonus was mainly multi-focal, affecting the upper and lower limbs in 88% (DA 190 and 181/272, respectively). The orofacial area and the trunk were less affected, in 63 and 54% (DA 141 and 131/272, respectively). Finally, the jerks were generalized in 59% (DA 137/272). Among patients with myoclonus affecting the limbs, jerks were more frequent over distal (69%) than proximal (31%) limbs (DA 29/272). A right-left asymmetry was noted in a few. The myoclonus was elicited by action in all patients (DA 211/272). This action myoclonus represented the most common clinical feature of LAS and the main cause of daily disability, strongly impairing the quality of life of patients. Action myoclonus dramatically increased with the precision of the motor tasks, as reported in Lance and Adams’ eloquent clinical description, making everyday tasks, such as bringing a cup to the mouth, grasping a small object, and dressing extremely difficult.^1^ Moreover, stimuli elicited myoclonus in 81% (DA 146/272). Somatosensory stimuli were the main activation mode of reflex myoclonus, found in 72%, followed by auditory stimuli in 52%, and visual stimuli in 13% (DA 105, 90, and 56/118, respectively). Myoclonus was present during rest in 47% (DA 154/272) and was also elicited by stress, anxiety, and emotional or surprising contexts in a few patients.

Negative myoclonus was reported only in 11% of patients included in this review, including the archetypal cases of Lance and Adams, mainly affecting lower limbs.^18^ However, in our recent study of 18 patients, negative myoclonus was found in 44% of them during the clinical exam and in 88% using polymyography of myoclonus.^8^ These postural lapses, occurring while standing or walking, contribute significantly to functional impairment, especially regarding their traumatic consequences and the induced apprehension of walking with the risk of falling, resulting in some patients being confined to wheelchairs.

### Other neurological symptoms — Cerebellar-labelled symptoms, cognitive impairments and seizures are the main accompanying symptoms

The main associated symptoms were cerebellar, reported in 55% (DA 110/272). They mainly consisted of walking and gait disturbance in 93% and hypermetric movements in 25% of them. However, myoclonus can disrupt movements so much that it seems very difficult to detect a cerebellar disturbance. In a large cohort of 18 patients extensively examined, we only identified 1 patient with a definite cerebellar syndrome,^8^ questioning the presence of such syndrome in previous reports. Secondly, cognitive deficits were also reported in 50% (DA 120/272). They mainly consisted of memory impairment in 33%, attention deficit in 18%, dysexecutive syndrome in 15%, and language impairment in 12% of patients with cognitive impairments. The presence or absence of seizures was reported in some patients (DA 140/272). Among the 57 patients with seizures, semiology was reported in 45 of them. Patients had only generalized seizures in 89%, focal seizures in 7%, and both generalized and focal seizures in 4%. Nine patients strikingly showed myoclonus decrease after spontaneous generalized seizures.^8,19,20^ In a few patients, other motor symptoms were reported such as pyramidal syndrome, extrapyramidal syndrome, motor deficit, or movement disorders other than myoclonus. Finally, disorders of consciousness were very rarely reported.

## Biological findings

### Blood explorations — Lance–Adams syndrome is not associated with any specific blood abnormalities

Standard routine blood sample results were available in 29 patients and were normal in most patients (complete blood count, kidney and liver function, standard metabolic and electrolyte panel). It rarely showed abnormalities related to the aetiology of anoxia or usual metabolic abnormalities in critically ill patients. The neuron-specific enolase between one to three days after the anoxic event was 27.2 (5.7–115) ng/ml (DA 18/272).

### CSF explorations — CSF compounds related to serotonin metabolism may show some defects

The CSF features, obtained within a wide acquisition delay among patients, were found in 26 patients. Standard cellular and biochemical analyses identified normal results in seven and hypo-proteinorachia and hyper-proteinorachia in one each (DA 9/272). Before any serotoninergic therapy, the tryptophan (precursor of serotonin) dosage was normal in all the tested patients and the 5-hydroxyindoleacetic acid (5-HIAA, main metabolite of serotonin) dosage was either normal in around half of the tested patients or decreased/undetectable in the others (DA 19/272). These low CSF levels of serotonin metabolites associated with the partial positive response to some serotoninergic treatment [such as 5-hydroxytryptophan (5-HTP), described in the below-dedicated section] may suggest the contribution of serotoninergic neurons’ damage or dysfunction in LAS. However, post-mortem human histological studies found no lesions in serotoninergic structures (see below). Some animal studies (not included in this review which is focused on human data) also showed a decrease in myoclonic jerks in rodents with PHM treated with 5-HTP and some agonists or antagonists of subtypes of serotonin receptors.^21-24^ However, results were discordant concerning the intracerebral dosages of 5-HTP, serotonin, and 5-HIAA in various brain structures of these rodents.^25,26^ Therefore, these findings seem non-specific or point to the possible and complex involvement of specific serotonin receptors.

## Anatomopathological findings

### Post-mortem human explorations — Lance–Adams syndrome is not associated with any obvious histopathological abnormalities

We only found two studies reporting post-mortem histology of patients with LAS that did not show similar results. One found severe widespread neuronal loss and astrocytic gliosis in basal ganglia, especially the sub-thalamus nucleus (STN) and the ventrolateral (VL) nucleus of the thalamus, and in the pontine reticular formation, and discrete lesions in the frontal cortex.^27^ The other study only showed limited lesions in the parieto-occipital cortex, putamen, and caudate nucleus.^28^

## Neurophysiological characteristics

We will first present the results of EEG recorded during coma in patients who will further develop LAS and then the results of EEG, polymyography of myoclonus and EEG–EMG coupled analyses with JLBA performed far from the coma period/after regaining consciousness for all patients with LAS.

### EEG during the coma period of patients who will further develop Lance–Adams syndrome — EEG may show poor prognosis patterns or even central midline epileptiform discharges

At least one EEG performed during coma was reported in 56/272 patients with LAS. The mean delay of this EEG acquisition was 2.0 (0.5–10) days (DA 50/56). The background activity was slowed in 59%, masked by a SE in 17%, normal in 15%, with burst suppression (BS) patterns in 7% and with alpha coma pattern in 2% of patients (DA 41/56). Epileptiform discharges (EDs) were found in 63% (DA 40/46), after the exclusion of those with SE and BS. These ED were localized in the central midline region in 80%, were generalized in 16% and were recorded in the bilateral frontal regions in 4% (DA 25/25). Among patients without SE and BS and with myoclonus during EEG, jerks were always associated with ED in 70% (DA 10/12). These findings suggest that EEG may provide valuable information precociously after anoxia as the typical central midline localization of ED may appear early in the natural history of patients with LAS, making it a precious tool for early prognostication.^29,30^ Moreover, it suggests that, although very rare, some patients with initially poor prognosis EEG patterns, such as BS or alpha coma, may survive and develop LAS.

### EEG after the coma period/regaining consciousness — Lance–Adams syndrome is associated with frontal and central median epileptiform discharges

**Figure 3:**
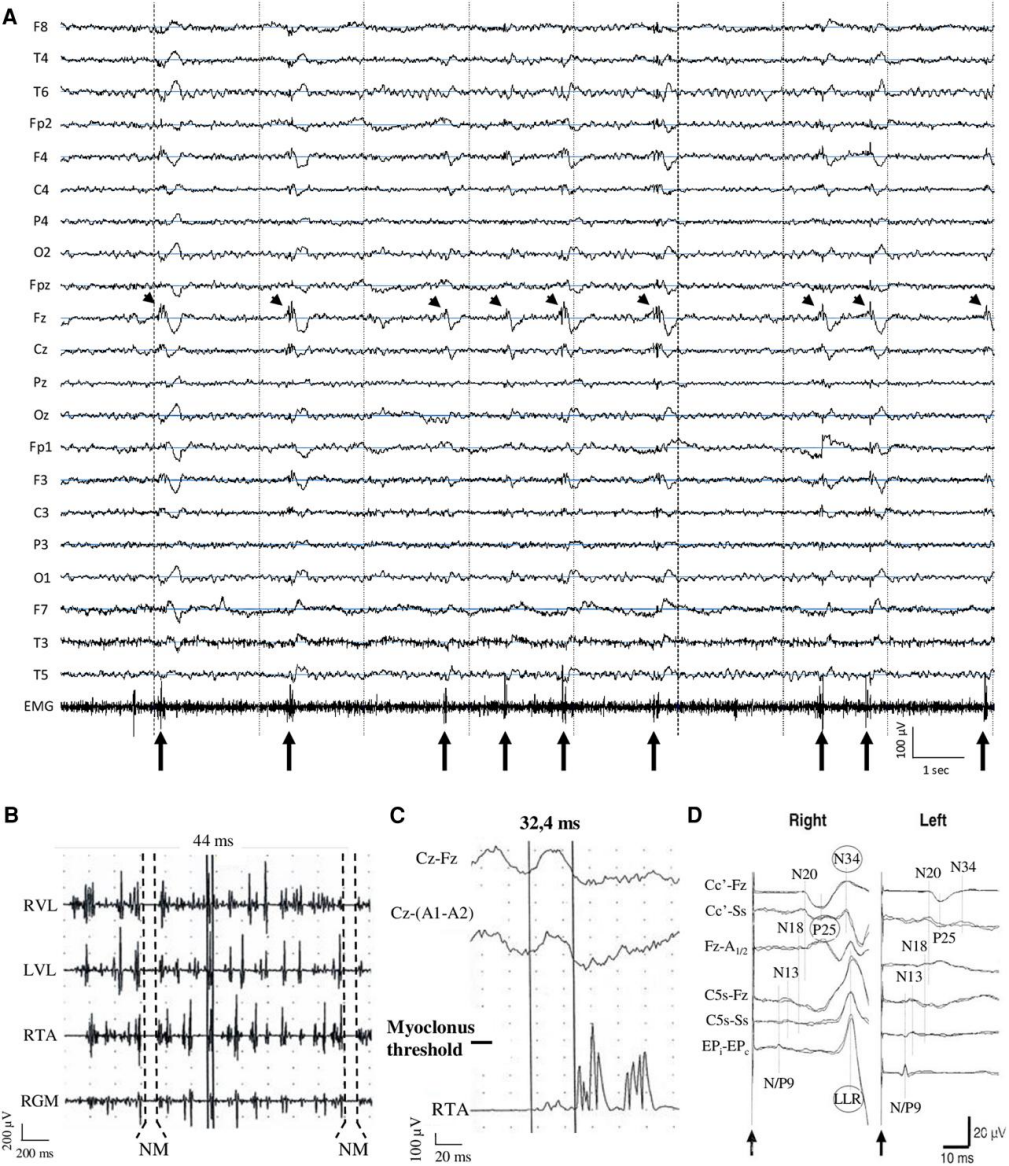
**Neurophysiological EEG and EMG explorations in patients with LAS.** (**A**) Scalp EEG (common average reference montage). EDs, here spikes-and-waves and polyspikes-and-waves (arrowheads), are located on the fronto-central regions with a predominance on the midline and are associated with myoclonic bursts (arrows). (**B**) Polygraphic lower limbs EMG recording. Traces from up to bottom represent recordings over the right vastus lateralis (RVL), left vastus lateralis (LVL), right tibialis anterior (RTA) and right gastrocnemius medialis (RGM). During orthostatism, short-duration muscular jerks (positive myoclonus, duration of 44 ms in the example highlighted with continuous lines) are associated with electrical silences [negative myoclonus (NM), duration >100 ms in the example highlighted with discontinuous lines]. (**C**) EEG JLBA (113 myoclonic jerks recorded over the right tibialis anterior muscle) in the same patient shows a pre-myoclonic cortical potential on the central median electrode with a delay of −32.4 ms. ((**A–C**) These are published in Vellieux *et al.*^20^ and reused with permission (Copyright Elsevier). (**D**) SSEPs (2 series of 250 responses are superimposed) following right (left column) and left (right column) nerve stimulation at the wrist. Traces from below to above represent recordings over the brachial plexus (N/P9; EP2–EP1; EP1–EP2), fifth cervical vertebra with a suprasternal and a frontal reference (N13; C5s–Ss; C5s–Fz), frontal cortex with a linked ear reference (N18; Fz–A1/2) and the hand area of the contralateral sensory cortex with a non-cephalic (suprasternal) and a frontal reference (N20, P25, N34; C3′–Ss; C4′–Ss; C3′–Fz; C4′–Fz). The arrows indicate stimulus onset. Following right median nerve stimulation (left column): (i) in the upper three traces, the cortical responses P25 and N34 are enlarged, and (ii) in the lower three traces, a long-loop reflex is recorded. This figure is published in Schauer *et al.*^31^ and reused with permission (Copyright John Wiley and Sons).

At least one EEG was performed far from the acute period in 146/272 patients, with a wide acquisition delay among patients. Overall, the midline electrodes were included in the EEG montage in 39% of patients whereas they were not included in 6%. This information was lacking in the remaining 55% of patients. We found that EDs were present in 54% of patients (DA 136/146). The ED were recorded over the frontal-central midline electrodes (Fz and Cz) and to a lesser extent frontal and central suprasylvian electrodes (F3, F4, C3 and C4), anatomically located on the motor cortical areas (Fig. 3A), or were generalized with central predominance in 82% (DA 60/73). A quantitative analysis of the ED amplitude peak confirmed this localization and showed a significant and positive correlation between the durations of ED and myoclonic jerks.^8^ For the other 46% of patients without reported ED, the midline electrodes’ recording was lacking in 75% of them. The EDs have probably been missed in these patients, especially if they are of low amplitude. We thus strongly recommend including midline electrodes, especially Fz, Cz and Pz, during the EEG recording of patients with myoclonus after anoxia to provide the most accurate diagnosis. In the majority of recordings, EDs were spikes and spike-and-waves. Polyspikes and polyspikes-and-waves were also present but less common. Interestingly, sleep revealed midline ED that were absent during wake in one patient^32^ and increased the frequency of ED compared with wake in others.^8^ On the contrary, sleep reduced ED in one patient.^33^ Among the 14 patients without any ED on the early EEG, ED were identified in two of the three patients who had a late EEG.^34,35^

Intracranial EEG stereotactic study was performed in one patient using 7 electrodes with a total of 90 recording contacts. Various (i) cortical and (ii) sub-cortical areas were recorded: (i) primary motor cortex, primary somatosensory cortex, Rolandic operculum, middle frontal cortex, paracentral lobule and supplementary motor area and (ii) VL nucleus of the thalamus, internal capsule, head of the caudate nucleus, putamen and pallidum.^19^ In the motor cortical area, a spike preceded each spontaneous myoclonic jerk of the feet, the face, or the tongue. The motor cortical spikes were followed, and never preceded, by spikes in the VL nucleus of the thalamus. The motor cortex stimulation triggered a myoclonic jerk in the corresponding muscular territory in a delay similar to the latency between the spontaneous spikes and jerks. Moreover, the motor cortex stimulation triggered a spike in the VL. Finally, the stimulation of the VL did not modify the cortical activity and did not determine myoclonus.

### Polymyography of myoclonus — Lance–Adams syndrome is mainly associated with short-duration muscular jerks and longer electrical silences

Polymyography was performed only in 56/272 patients with most of the time very little available data. The main information that could be extracted from polymyography was the duration of myoclonic jerks available in 27 patients. It extended from 20 to 200 ms during action (DA 22/27), 10 to 50 ms during rest (DA 9/27) and 20 to 400 ms in response to stimuli (DA 11/27), but was <50 ms in most cases (Fig. 3B). The topographic muscular activation pattern was rarely reported. In most of the patients, a down-descending pattern suitable with corticospinal conduction was observed,^8,36^ whereas few showed an up-ascending pattern of myoclonus.^36-39^ Electromyographic electrical silences, corresponding to the clinical negative myoclonus as the cessation of muscular activity, were recorded in 25 patients and lasted from 30 to 500 ms (Fig. 3B), always following positive myoclonus,^8^ and simultaneous with the slow waves of the EEG spike-and-waves.^40^

### Coupled EEG–EMG analyses with jerk-locked back averaging — Myoclonic jerks in Lance–Adams syndrome are associated with a pre-myoclonic cortical potential

Coupled EEG–EMG analyses with the JLBA method were reported only in 49/272 patients. A time-locked pre-myoclonic potential over the contralateral or median sensorimotor cortical area was found in 90% of patients (DA 49/49) (Fig. 3C). Interestingly, 93% of the patients with no ED on the late EEG showed a time-locked pre-myoclonic cortical potential (DA 14/63).

The absence of identification of a pre-myoclonic potential does not mean that myoclonus is not of cortical origin. First, the cortical event amplitude may be too low to be registered considering the EEG background activity.^41^ Second, when cortical myoclonus occurs rhythmically, the JLBA method may fail to detect any significant cortical activation before the jerks or may dramatically attenuate its amplitude or modify its shape, thereby reducing the sensitivity and specificity of the technique.^42^

### Evoked potentials findings — Lance–Adams syndrome is sometimes associated with giant somatosensory evoked potentials and exaggerated long latency reflexes

Results of SSEP and LLR studies were available only in 29 and 15 patients respectively and showed giant SSEP in 28% and an exaggerated transcortical long-loop reflex in 27% (Fig. 3D). The major bias of these findings is the concomitant use in many patients of one or more anti-seizure medications (ASMs), which can decrease cortical hyper-excitability and modify functional explorations of cortical excitability.^43^

Finally, one patient was explored with paired-pulse transcranial magnetic stimulation.^44^ It showed a decrease in the amplitude of short intra-cortical inhibition and an increase in the amplitude of long intra-cortical inhibition and intra-cortical facilitation, raising the role of a decreased intra-cortical gamma-aminobutyric acid (GABA) inhibition in cortical pathology.

## Brain imaging findings

### Morphologic MRI — Lance–Adams syndrome is not associated with any specific morphologic abnormalities

Findings from morphologic brain MRI were available in 111/272 patients. We will first present the results of brain MRI during the acute period in patients who will further develop LAS and then the results of brain MRI performed far from the acute period/after regaining consciousness for all patients with LAS.

The MRI was performed during the acute period in 41 patients with a delay of 6 (0–26) days after the anoxic event (DA 29/41). It was described as normal/with no acute lesions in 59% and abnormal/with acute lesions in 41% of cases. Among the 17 patients with an abnormal MRI during the acute period, imaging found, in a non-mutually exclusive way, mainly signal abnormalities related to hypoxic lesions in diffusion-weighted images (DWI) and/or T2/T2-Fluid attenuated inversion recovery (FLAIR) and/or T1 in basal ganglia in 59% (in the striatum, lenticular nucleus, putamen, globus pallidus or without anatomical details), thalami in 29% (in pulvinar or without anatomical details), cortex in 24% (in frontal, perirolandic, parietal, insular or occipital areas) and cerebellum in 6%.

A morphologic brain MRI was performed far from the acute period/after regaining consciousness in 60/111 patients, with a wide acquisition delay among patients. It was described as normal in 33% and abnormal in 67% of cases. Among the 40 patients with an abnormal MRI far from the acute period, imaging found, in a non-mutually exclusive way, mainly atrophy in 53% of cases (diffuse in almost all cases) and various signal abnormalities in 28% of patients. In most cases, hyper-/hypo-intensities were located in sub-cortical structures: basal ganglia, white matter (WM) with various localizations (including the pyramidal tract) and brainstem. Cortical structures were more rarely affected. Finally, 25% of patients also had strokes, lacuna or non-specific microvascular ischaemic changes. We found very few patients who benefited from iterative brain MRI that revealed different evolutionary profiles: no signs of hypoxic encephalopathy,^8,45,46^ regression of cerebellar and thalamic hyper-intensities,^47^ emergence of hyper-intensities in sub-cortical structures,^48-50^ persistence of basal ganglia hyper-intensities with appearance of atrophy^48^ or emergence/increase of brain atrophy.^20,51^

### Functional explorations — Heterogeneous brain metabolic patterns are found in Lance–Adams syndrome but motor and pericentral hypometabolism seems predominant

**Figure 4:**
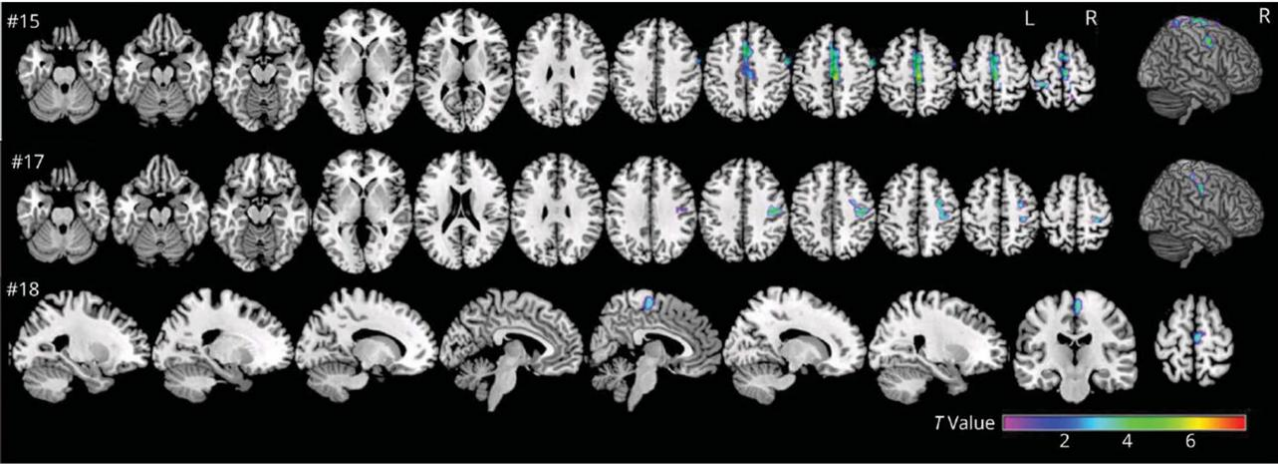
**Brain ^18^F-FDG PET/CT imaging in patients with LAS.** The colour scale (T-value) shows regions with glucose hypometabolism in patients with LAS versus 55 healthy controls, on a projection onto T1-weighted MR images. Significant hypometabolism is found in the motor and pericentral cortical areas: pre-central gyrus (upper and middle parts), post-central gyrus (upper part), paracentral lobule (upper part) and supplementary motor area (upper and lower parts). L, left; R, right. This figure is published in Vellieux *et al.*^8^ and reused with permission (Copyright Wolters Kluwer Health Inc.).

Functional brain imaging was performed in 27/272 patients. The 22 PET exams were performed in a wide range of delays after the anoxic event, from 51 days to 25 years. Results were abnormal for all and showed various patterns of hypo and hyper-metabolism. An increase in glucose metabolism was found in bilateral medial temporal lobes, pontine tegmentum, mesencephalon and VL nucleus of the thalamus in a series of seven patients.^52^ Moreover, a decrease in glucose metabolism was found in a few other patients in different cortical and sub-cortical regions, making it difficult to highlight a hypometabolism pattern in LAS. However, our recent cohort study including PET data in ten patients showed a significant and common pattern of hypometabolism in the motor and pericentral areas using volume-of-interest and voxel-wise analyses (Fig. 4).^8^ Five SPECT exams were performed from 20 to 330 days after the anoxic event. One exam was normal, and the others showed various patterns of hypo- and hyper-perfusion in various brain cortical areas, basal ganglia and cerebellum, but without any common pattern.

A fMRI was performed in two patients: increase in blood oxygenation level dependent (BOLD) effect of bilateral cortical areas (particularly the legs’ motor cortex) more robustly enhanced with action for one (delay between anoxia and fMRI not available),^53^ and increased functional connectivity between motor and supplementary motor cortex to various parietal and temporal cortical areas compared with controls for the other (fMRI performed 40 days after anoxia).^35^

MRS was reported in seven patients showing normal results^8^ or various patterns of spectroscopic changes: mild to moderate reduction of N-acetyl-aspartate (NAA) in both hippocampi,^54^ decrease of the NAA/creatine ratio in the posterior cingulate gyrus and the parietal WM,^51^ or moderate decrease of NAA in the right thalamus and the left insular cortex.^8^

Finally, one patient was explored using diffusion tensor imaging 6 months after anoxia.^44^ It showed a decrease in the fractional anisotropy and an increase in the mean diffusivity (MD) in the pediculopontine nucleus and an increase in the MD of the right red nucleus, thalamus, and left STN compared with controls.

## Discussion on the localization of the myoclonus generator

The myoclonus generator’s localization was rarely assessed by the authors of the studies included in this work (DA 64/272). According to the authors who reported this data, myoclonus was considered cortical in 80%, cortical and sub-cortical in 9%, sub-cortical reticular in 8% and associated with another mechanism in 3%. Myoclonus was of cortical origin in the vast majority of patients with LAS and arguments were as follows: (i) multi-focal and distal topography of myoclonus, (ii) ED on EEG recordings, (iii) typical cortical profile of myoclonic jerks on polymyography, (iv) pre-myoclonic EEG potential on EEG–EMG coupled analyses with JLBA, (v) giant SSEP and (vi) exaggerated LLR. Arguments for the sub-cortical origin of myoclonus in the few other patients were as follows: (i) more generalized or proximal topography of myoclonus and reflex or rest myoclonus, (ii) absence of ED or ED not always time-locked with myoclonic jerks on EEG recordings, (iii) myoclonic jerks’ sub-cortical profile on polymyography, (iv) absence of pre-myoclonic EEG potential on EEG–EMG coupled analyses with JLBA and (v) normal SSEP. Except for the sporadic patients with a sub-cortical profile on polymyography, all the other arguments put forward for the sub-cortical origin of myoclonus may be debatable. First, the accurate clinical diagnosis of the subtype of myoclonus remains challenging. Myoclonus’ clinical features alone may have led to mistakes in subtyping the anatomical classification of myoclonus.^55^ Second, as discussed above, some authors may have missed ED on EEG recordings if midline electrodes were not recorded in patients with cortical myoclonus due to LAS. Moreover, neurophysiological investigations are needed to supply confirmatory evidence for a cortical origin of myoclonus, especially for detecting pre-myoclonic EEG potential on JLBA, giant SSEP and exaggerated LLR. However, not all of these three key neurophysiological features are systematically present in patients with cortical myoclonus.^56^ The diagnostic accuracy of cortical myoclonus can be increased only by combining observations from multiple tests.^57^

In addition, the main central midline localization of ED and the hypometabolism pattern of motor and perirolandic cortical areas in some PET studies suggest that myoclonus in LAS may originate from the motor cortex.

## Treatment

### Pharmacological interventions — Benzodiazepines and some anti-seizure medication decrease myoclonus

**Figure 5:**
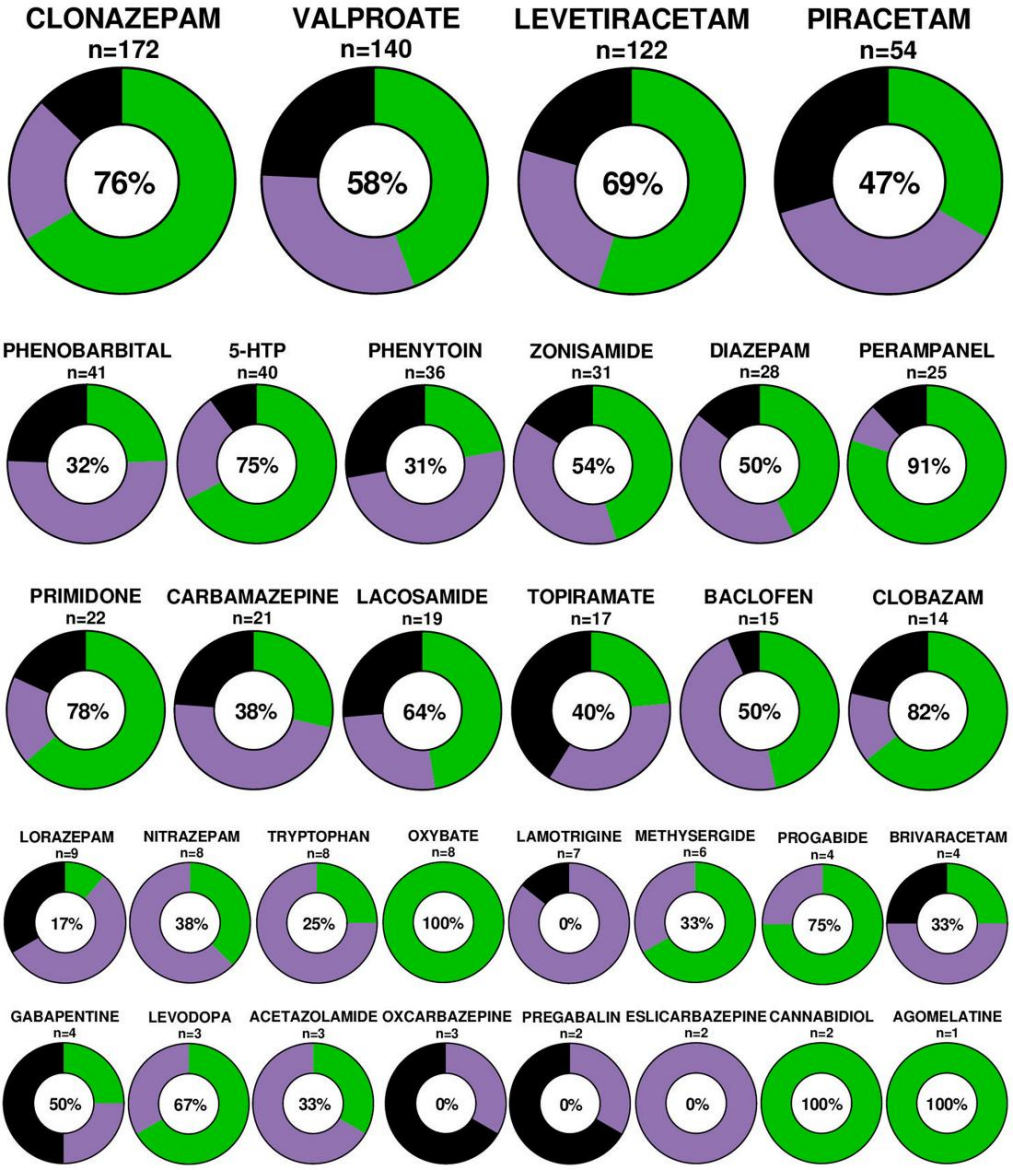
**Summary of the effects of reported pharmacological interventions.** The green part represents the proportion of patients who showed at least mild efficacy, the violet part represents the proportion of patients with no efficacy and the black part represents the proportion of patients with no available efficacy data. The number of patients in whom the treatment was tried (*N*) and the positive effect ratio [(green/green + violet), %] are presented for each molecule. The first line represents molecules used in at least 50 patients (large circles), the middle lines represent molecules used in 10–50 patients (medium-sized circles) and the bottom lines represent molecules used in <10 patients (small circles).

Many pharmacological treatments, mainly ASM, have been tried in patients with LAS. No large randomized controlled treatment trials are available. Therapeutic effects were always reported in descriptive terms giving only an approximation of the drugs’ effectiveness. Thus, we must be cautious concerning the precision of the data extracted from the included studies. Details concerning the use and positive effect on myoclonus of the most prescribed treatments are provided in Fig. 5. The three most commonly used drugs, in more than a hundred patients each, were clonazepam, valproate and levetiracetam, each with a positive effect ratio (among the patients with available efficacy data) of 76, 58 and 69%. Piracetam improved almost half of the patients. We also identified reports suggesting high positive effect ratios of 5-HTP (75%), the immediate precursor of serotonin, from studies mainly published in the 70s and 80s, and oxybate (100%), the sodium salt of gamma-hydroxybutyrate, which is a derivative of gamma-aminobutyric acid, from studies published during the 2000s and 2010s. Patients whose myoclonus decreased with oxybate were also alcohol-responsive. More recently, a positive effect on myoclonus was reported with perampanel and lacosamide, in 91 and 64% of patients. However, these later findings rely on the reports of a small number of patients.

Some drugs worsened myoclonus in a few patients: haloperidol, levopromazine, chlorpromazine, dihydroergotamine, tryptophan, 5-HTP, methysergide, valproate and lamotrigine. We did not find any reports mentioning the worsening of myoclonus with sodium channel blockers like carbamazepine or phenytoin, whereas it is a common finding in our opinion.

Besides their clinical positive effects, clonazepam,^36,40,58,59^ diazepam,^59^ levetiracetam,^60^ valproate^61^ and 5-HTP^62,63^ were reported to reduce ED and even normalize EEG recordings in a few patients. In one case, levetiracetam was also reported to suppress the jerk-locked cortical responses on JLBA.^31^ Moreover, in very few patients, some authors reported that the therapy with 5-HTP induced an increase in the CSF levels of 5-HTP, serotonin, or 5-HIAA,^62-65^ the therapy with valproate an increase/normalization of the CSF 5-HIAA level^66^ and the therapy with both 5-HTP and valproate an increase in the CSF levels of tryptophan and 5-HIAA.^67^

In addition, we did not identify enough findings on drug tolerance and long-term retention, except our work which showed that levetiracetam, valproate, clonazepam, zonisamide, and perampanel had better long-term retention rates than those of other drugs.^8^

Nevertheless, many patients with LAS continue to present severe myoclonus and disability despite the combination of ASM.

### Non-pharmacological interventions — Some invasive or non-invasive neuromodulation techniques may improve patients with Lance–Adams syndrome

Non-pharmacological approaches were reported in medication-refractory patients. Two patients, including the one who benefited from a stereotactic study previously described, had unilateral destruction of the VL nucleus of the thalamus that did not show any clear efficacy.^19,68^ Ten patients, reported in as many case reports from different teams, benefited from deep brain stimulation (DBS), all with bilateral globus pallidus internus (GPi) stimulation,^69-76^ except one with unilateral GPi stimulation^77^ and one with bilateral STN stimulation.^78^ According to the authors, three had cortical myoclonus, one had cortical and sub-cortical myoclonus, and one had non-cortical myoclonus. The assumed localization of the myoclonus generator for the five others was not specified. The DBS showed a decrease, even suppression, of myoclonus in seven of these patients, according to the Unified Myoclonus Rating Scale,^79^ especially the action myoclonus section, assessed before and after implantation. This clinical improvement allowed a reduction in ASM therapy in some cases. In two other patients, DBS was ineffective. For the last one, the result of DBS was not reported. Interestingly, one of the patients who showed an improvement of myoclonus with bilateral GPi DBS had frequent generalized ED on the EEG recorded before DBS. The EEG performed five years after the DBS implantation did not show any ED (with stimulation off).^76^

Finally, we recently reported the major improvement of cortical action myoclonus in a patient with refractory LAS using iterative electroconvulsive therapy, to reproduce spontaneous generalized seizures that also led to myoclonus decrease.^20^ It may represent a promising treatment that should be further evaluated in some selected patients with LAS.

## Conclusion

Our review described the specific clinical and neurophysiological features of LAS, together with non-specific imaging and biological findings, and stressed the different therapeutical options. Most patients with LAS suffered from a primary hypoxic event. Some patients with LAS experienced myoclonus very soon after anoxia, before awakening, with a semiology quite different as myoclonus was more likely to be generalized and stimuli-induced during the coma period than after awakening. Early EEG recordings during coma might already show the LAS characteristic pattern of frontal/central midline EDs, which may help intensivists refrain from withdrawing life-sustaining therapies for patients with myoclonus after anoxia.

**Figure 6:**
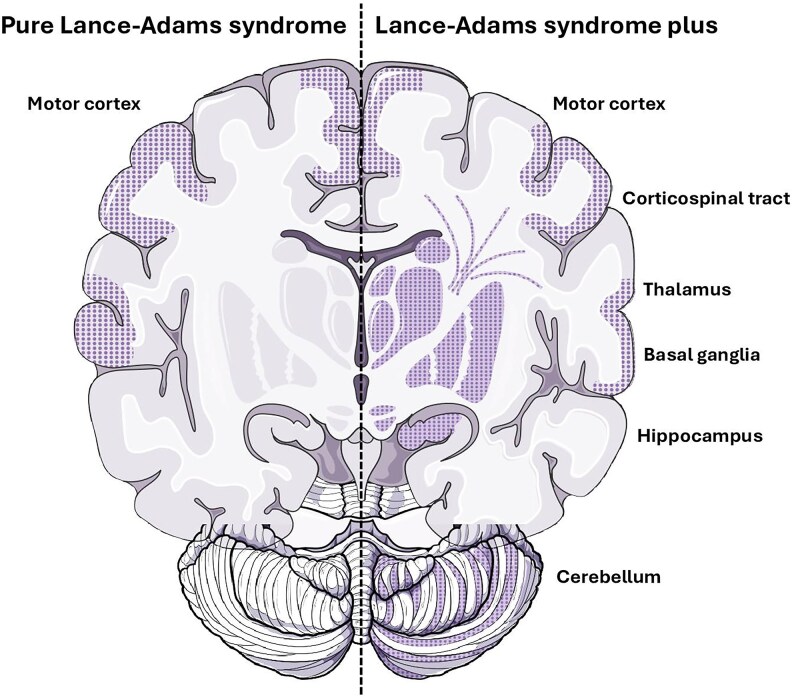
**Schematic representation of the extent of anoxia leading to LAS.** On the left side, pure LAS is related to restricted and focal post-hypoxic hyper-excitability of the motor cortex with no or minimal other brain lesions. It leads to action or reflex, multi-focal or generalized, myoclonus predominant to distal limbs and face, with no or minimal other neurological symptoms. On the right side, LAS plus is related to widespread significant brain post-hypoxic lesions, including the motor cortex and other cortical and sub-cortical structures such as the corticospinal tract, thalamus, basal ganglia, hippocampus or cerebellum. It leads to action myoclonus associated with significant and disabling other neurological symptoms, such as pyramidal syndrome, extrapyramidal syndrome, other movement disorders, memory impairment or cerebellar ataxia. Hatched areas represent the post-hypoxic lesions, dark grey is for grey matter and light grey is for WM.

Myoclonus of patients with LAS mainly consists of action-induced multi-focal myoclonic jerks predominant to distal limbs and face and has overwhelmingly the features of cortical myoclonus. The localization of EEG EDs and brain imaging metabolic patterns suggest that this cortical myoclonus originates within the motor cortex. Thus, we may conclude that the chronic post-hypoxic multi-focal action-induced myoclonus with electrophysiological features of a cortical generator is the main form of LAS and is related to a restricted and focal hyper-excitability of the motor cortex. This condition may be referred to as ‘pure LAS’. Moreover, some patients may present, in addition to PHM, other symptoms (i.e. pyramidal, extrapyramidal or definite cerebellar syndrome, disorders of consciousness, cognitive deficit or others) related to broader brain post-hypoxic lesions. This condition may be called ‘LAS plus’ (Fig. 6). Thorough clinical, electrophysiological and neuroradiological explorations may help differentiate between these two conditions. Patients with pure LAS, who remain severely disabled despite adjusted ASM, may benefit from promising neuromodulation techniques that can have a significant effect on myoclonus-related disability.

## Supplementary Material

fcaf329_Supplementary_Data

## Data Availability

The data supporting the findings of this study are available from the corresponding author upon reasonable request.
